# Mitochondrial Division Inhibitor 1 Attenuates Mitophagy in a Rat Model of Acute Lung Injury

**DOI:** 10.1155/2019/2193706

**Published:** 2019-05-08

**Authors:** Xu Luo, Ruimeng Liu, Zhihao Zhang, Zhugui Chen, Jian He, Youtan Liu

**Affiliations:** ^1^Department of Critical Care Medicine, People's Hospital of Longhua, Shenzhen 518109, China; ^2^Department of Anesthesiology, Shenzhen Hospital, Southern Medical University, Shenzhen 518110, China; ^3^Department of Anesthesiology, Affiliated Hospital of Guangdong Medical University, Zhanjiang 524023, China; ^4^Department of Anesthesiology, The First People's Hospital of Foshan, Foshan 528000, China

## Abstract

The regulation of intracellular mitochondria degradation is mediated by mitophagy. While studies have shown that mitophagy can lead to mitochondrial dysfunction and cell damage, the role of Mdivi-1 and mitophagy remains unclear in acute lung injury (ALI) pathogenesis. In this study, we demonstrated that Mdivi-1, which is widely used as an inhibitor of mitophagy, ameliorated acute lung injury assessed by HE staining, pulmonary microvascular permeability assay, measurement of wet/dry weight (W/D) ratio, and oxygenation index (PaO2/FiO2) analysis. Then, the mitophagy related proteins were evaluated by western blot. The results indicated that LPS-induced activation of mitophagy was inhibited by Mdivi-1 treatment. In addition, we found that Mdivi-1 protected A549 cells against LPS-induced mitochondrial dysfunction. We also found that Mdivi-1 reduced pulmonary cell apoptosis in the LPS-challenged rats and protected pulmonary tissues from oxidative stress (represented by the content of superoxide dismutase, malondialdehyde and lipid peroxides in lung). Moreover, Mdivi-1 treatment ameliorated LPS-induced lung inflammatory response and cells recruitment. These findings indicate that Mdivi-1 mitigates LPS-induced apoptosis, oxidative stress, and inflammation in ALI, which may be associated with mitophagy inhibition. Thus, the inhibition of mitophagy may represent a potential therapy for treating ALI.

## 1. Introduction

Clinical acute lung injury (ALI) is a common complication that occurs following sepsis among ICU patients and is associated with a high morbidity and mortality [1, 2], for which there are currently no biological therapies [3]. LPS is a major endotoxin component of gram-negative bacteria and plays an essential role in the development of ALI [4, 5]. Currently, it is thought that the multiple organ dysfunctions associated with sepsis can be attributed to a pathochemical and pathophysiological injury cascade, including the inflammatory response, macroautophagy, mitochondria dysfunction, and apoptosis [6, 7]; moreover, oxidative stress is also involved in the pathogenesis of ALI [8].

The lysosomal machinery is used to remove dysfunctional mitochondria through selective degradation via autophagy, a process termed mitophagy. However, the role of mitophagy in the development of disease remains controversial. While some studies have confirmed that excessive mitophagy promotes mitochondrial damage in chronic obstructive pulmonary disease and excessive mitochondrial fission is related to neurodegeneration and other mitochondriopathies [9, 10], its role in ALI remains unknown. It has been demonstrated that mitochondrial dynamics, which are primarily regulated by the GTPase dynamin-related protein, play a crucial part in regulating mitophagy [11, 12]. Mitophagy can be blocked using the mitochondrial division inhibitor 1 (Mdivi-1).

Thus, previous studies have shown that excessive mitophagy can cause mitochondrial damage and that Mdivi-1 can provide protection against various pathological conditions [13]. In this study, we hypothesize that mitophagy contributes to the development of ALI, whereas treatment with Mdivi-1 may prevent LPS-induced mitophagy and alleviate lung injury in rats. Our findings suggest that the inhibition of mitophagy may merit further exploration as a potential therapy for the treatment of ALI.

## 2. Materials and Methods

### 2.1. Reagents and Antibodies

MitoProbe™ JC-1 (5,5′,6,6′-Tetrachloro-1,1′,3,3′-tetraethyl-imidacarbocyanine iodide) were purchased from Molecular Probes (Invitrogen, CA, USA). The CellTiter-Glo® assay and a terminal deoxynucleotidyl transferase dUTP nick-end labeling (TUNEL) staining kit were supplied from Promega Corp. (Madison, WI, USA). Human lung alveolar epithelial cell lines (A549) were obtained from Guangzhou Cellcook Biotech Co., Ltd. (Guangzhou, China). Caspase 3 activity assay kit was obtained from Biovision (San Francisco, USA). Immunohistochemical kits were provided by EnVision™ (Dako, Copenhagen, Denmark). LPO kit was obtained from Cayman Chemical Co. (Ann Arbor, Michigan, USA). SOD and MDA kits were obtained from Jiancheng (Nanjing, China). LPS (*E. coli* serotype O111:B4), Mdivi-1, and other chemicals were purchased from Sigma-Aldrich (Saint Louis, MO, USA).

### 2.2. Animals

Procedures involving animals and their care were approved by the Medical Faculty Ethics Committee of Southern Medical University, Guangzhou, China, and complied with the NIH Guidelines for the Care and Use of Laboratory Animals. Male Sprague-Dawley rats (weight: 180 g-220 g) were acquired from the Experimental Animal Centre of South Medical University and housed under temperature- and humidity-controlled conditions on a 12/12 h day/night cycle with unrestricted access to standard diet and tap water.

### 2.3. ALI Model

A rat model of ALI was induced by an intratracheal administration of LPS. Animals were anesthetized under sodium pentobarbital (30 mg/kg body weight) intramuscularly and then placed in a supine position. The trachea was surgically exposed, after which 5 mL of LPS (10 mg/kg body weight, Sigma) was slowly injected into the trachea of each rat.

### 2.4. Cell Culture and Stimulation

A549 cells were grown at 37°C in 5% CO_2_ in Dulbecco's modified minimum essential medium (DMEM) containing low glucose, penicillin (100 U/mL), streptomycin (100 U), and 10% fetal bovine serum. The cells were stimulated in 10 *μ*g/mL LPS for 6 h to establish LPS-induced ALI* in vitro*.

### 2.5. Drug Treatment and Experimental Grouping

The cells were randomly divided into 4 groups (n = 6 per group) using a random number table: (1) control group (cells treated with vehicle and without LPS stimulation); (2) Mdivi-1 control group (cells treated with 10*μ*M of Mdivi-1 and without LPS stimulation); (3) LPS group (cells pretreated with vehicle 60 min, followed by LPS stimulation); (4) Mdivi-1 group (cells pretreated with 10*μ*M of Mdivi-1 60 min before LPS stimulation). DMSO was used as vehicle.

The animals were randomly divided into 4 groups (n = 6 per group) using a random number table: (1) control group (pretreated with 0.5 mL DMSO for 60 min via the caudal vein and given 0.5 mL intratracheal normal saline (NS)); (2) Mdivi-1 control group (rats pretreated with a dose of Mdivi-1 (3 mg/kg) dissolved in 0.5 mL DMSO for 60 min and given 0.5 mL intratracheal NS); (3) LPS group (rats pretreated with 0.5 mL DMSO for 60 min via the caudal vein followed by LPS instillation); (4) Mdivi-1 group (rats pretreated with a dose of Mdivi-1 (3 mg/kg) dissolved in 0.5 mL DMSO for 60 min via the caudal vein followed by LPS instillation).

### 2.6. Western Blot Analysis

Proteins were resolved on 10% SDS-PAGE and electroblotted onto a polyvinylidene fluoride (PVDF) membrane and blocked in 5% skimmed milk. The membranes were incubated overnight at 4°C with primary antibodies (all antibodies were obtained from Abcam, Cambridge, UK) against TOM20 (1:1,000 dilution), TIM23 (1:1,000 dilution), PGC-1*α* (1:1,000 dilution), mt-TFA (1:1,000 dilution), LC3 (1:1,000 dilution), P62 (1:1,000 dilution), F4/80 (1:1000 dilution), and GAPDH (1:5,000 dilution), respectively. Horseradish peroxidase-conjugated antirabbit IgG (1:5,000 dilution) was used as the secondary antibody. Immunoreactivity was detected with an enhanced chemiluminescence detection system (Beyotime, Haimen, China) and visualized on X-ray film (Kodak, Shanghai, China).

### 2.7. Measurement of Mitochondrial Function

The mitochondrial membrane potential (ΔΨm) was determined using the potential-sensitive fluorescent dye JC-1. Cells were treated and subjected to LPS. JC-1 (5 *μ*M) was loaded onto cells for 15 min at 37°C. The results were visualized using an inverted fluorescent microscope (Nikon, Ti-E Live Cell Imaging System, Japan).

Intracellular ATP was determined by a luciferase-based assay (CellTiter-Glo®, Promega, Madison, WI), according to the manufacturer's recommendation. The luminescence was recorded in an automatic microplate reader (SpectraMax M5; Molecular Devices, Sunnyvale, CA, USA).

### 2.8. Immunohistochemistry

Bax and Bcl-2 expression in the lungs were visualized using commercial immunohistochemistry kits. The working dilution of the antibodies against Bax and Bcl-2 was 1:200.

### 2.9. Oxygenation Index (PaO_*2*_/FiO_*2*_) Analysis

Twelve hours following LPS administration, arterial blood was collected from the carotid artery and analyzed using a commercial blood gas analyzer (model ABL8000; Radiometer Copenhagen, Westlake, Ohio). The oxygenation index was determined as the ratio of arterial oxygen partial pressure/fractional inspired oxygen (PaO_2_/FiO_2_).

### 2.10. H&E Staining and Scoring

Twelve hours following the administration of LPS, the right lobes of the lungs were immersed in 10% neutral buffered formalin, fixed, paraffin-embedded, and sliced. Following H&E staining, pathological changes of the lung tissues were evaluated as in previous study [14].

### 2.11. Lung Microvascular Permeability Analysis

The permeability assay was performed as previously described [15]. A sample of the lung tissue was weighed, homogenized, and immersed in N,N-dimethylformamide (Sigma, USA). Eluted EB was measured at a wavelength of 620 nm using an automatic microplate reader and the amount was expressed as micrograms per 100 mg of dry tissue.

### 2.12. Lung Wet/Dry (W/D) Ratio In Vivo

Following the administration of LPS, the water content of the lungs was measured. The right lungs were excised, blotted, and weighed to obtain the wet weight, then desiccated at 80°C for 48 h to obtain the dry weight. The wet/dry ratio was calculated as an assessment of tissue edema.

### 2.13. Assessments of Cell Apoptosis

Apoptosis was measured using terminal deoxynucleotidyl transferase-mediated dUDP nick-end labeling (TUNEL) staining. The level of caspase 3 activity was determined in the lung tissue homogenates using a caspase 3 Fluorometric Assay Kit.

### 2.14. Measurement of Lipid Peroxides (LPO), Superoxide Dismutase (SOD), and Malondialdehyde (MDA)

The level of LPO, SOD, and MDA in lung was determined using commercial assay kits in accordance with the respective manufacturer's guidelines.

### 2.15. Measurement of Inflammatory Mediators in Lung and the Bronchoalveolar Lavage Fluid (BALF)

BALF samples were collected by washing the lung three times with 4 mL PBS through a tracheal cannula placed into each rat under anesthesia. Briefly, animals were sacrificed and the chest was opened, a median sternotomy was performed, and the trachea was isolated using a blunt dissection. Next, a suitable small-caliber tube was inserted into the airway and secured. Then PBS solution was infused slowly into the lungs, and the BALF was withdrawn into the tube. The fluid recovery rate was > 80%. Lavage samples were centrifuged at 1,500 g for 10 min at 4°C.

The concentration of TNF-*α*, IL-1*β*, and IL-6 in the lung and BALF was measured using a commercial enzyme-linked immunosorbent assay kit. The results were expressed as g/mg of tissue or pg/mL of BALF.

### 2.16. Myeloperoxidase Activity Assay

Lung tissues were harvested, rinsed, homogenized, and centrifuged. Supernatants were collected and subjected to enzyme-linked immunosorbent assay for determination of myeloperoxidase (MPO) activity using the MPO activity colorimetric assay kit (Biovision, Zurich, Switzerland).

### 2.17. Statistical Analysis

Experimental results were presented as the mean standard error (± SD). Statistical analysis was performed using a one-way analysis of variance (ANOVA) followed by an LSD multiple comparison test and Student's* t*-test where appropriate. A value of P < 0.05 was considered statistically significant, and n represents the number of animals.

## 3. Results

### 3.1. Mdivi-1 Treatment Attenuates Lung Injury following LPS Administration in Rats

**Figure 1:**
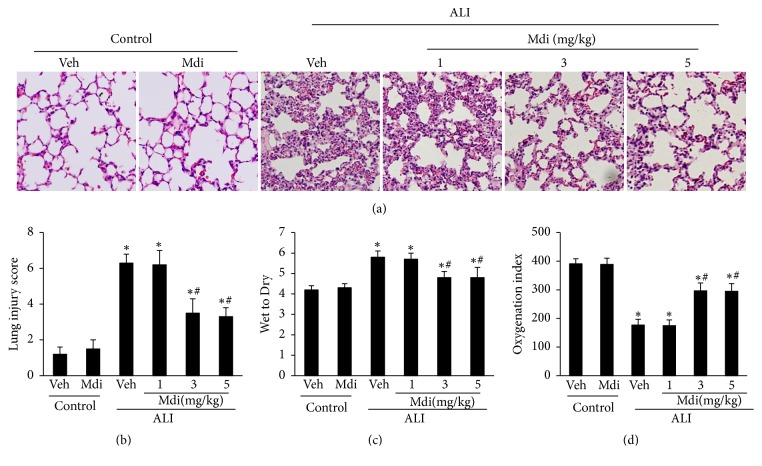
*Mdivi-1 treatment attenuates lung histopathology and pulmonary function*. (a) Comparison of the morphological changes of the lung tissues (200 × magnification). Hematoxylin and eosin staining: the LPS + vehicle group displayed severe inflammatory cell infiltration, diffuse edema with less alveolar space, and increased interstitial congestion. Moderate (3 mg/kg) and high (5mg/kg) doses of Mdivi-1 treatment markedly prevented the morphological changes induced by LPS administration. (b to d) Increased lung injury score and lung W/D ratio, as well as decreased PaO_2_/FiO_2_, were observed in the LPS + vehicle group, which were improved following both the moderate or high dose of Mdivi-1 treatment. Data are expressed as the mean ± SD (n = 6 in each group). *∗*P < 0.05 versus control group; #P < 0.05 versus LPS + vehicle group.

It has been well-established that the administration of LPS causes injury to the lung. In order to assess whether Mdivi-1 treatment was actually provided a protective role against acute lung injury in rats, H&E staining was performed on the lung tissues to observe the extent of injury within the lung. As shown in Figure 1(a), the LPS + vehicle group displayed severe inflammatory cell infiltration, thickened alveolar walls, diffuse edema, decreased alveolar space, and enhanced interstitial congestion compared to the control and vehicle groups, which were comparable. In addition, LPS exposure was also associated with an increased lung injury score and W/D ratio, as well as a decreased oxygenation index (P < 0.05 for LPS + vehicle versus control; Figures 1(b)–1(d)). These data suggest that the intratracheal administration of LPS results in significant ALI. Conversely, treatment with moderate (3 mg/kg) and high (5 mg/kg) doses of Mdivi-1 dramatically attenuated the observed histopathological changes, lung injury score, W/D ratio, and oxygenation index, whereas a small (1 mg/kg) dose could not achieve such effects (P < 0.05 for LPS + vehicle versus LPS + 3 mg/kg and LPS + 5 mg/kg; P > 0.05 for LPS + vehicle versus LPS + 1 mg/kg; Figure 1). So, we selected the dose of 3 mg/kg of Mdivi-1 as the treatment in the following experiment* in vivo*. Moreover, we found that Mdivi-1 significantly reduced the lung microvascular permeability reflecting by the lower content of Evans Blue in the lung (Figure 6(h)). These results demonstrate that Mdivi-1 treatment significantly improves the lung function in LPS-challenged rats.

### 3.2. Mdivi-1 Treatment Inhibits the Activation of Mitophagy in the Lung

**Figure 2:**
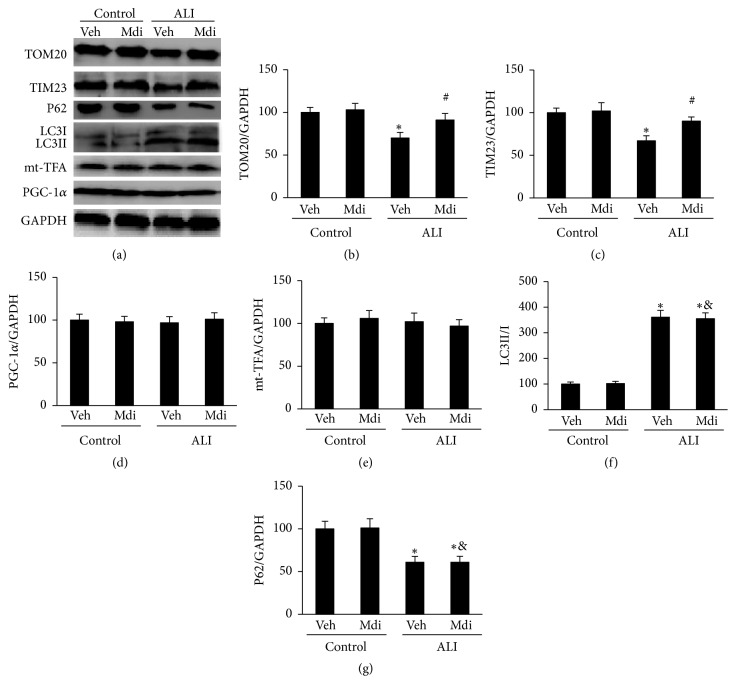
*Mdivi-1 treatment inhibits the mitophagy activation*. Mdivi-1 treatment prevented the LPS-induced loss of mitochondrial mass without influencing mitochondrial biogenesis and overall autophagy. (a) representative images of western blot results are shown; (b to g) semiquantitative analysis of protein levels. The values are expressed as the mean ± SD (n = 6 in each group). *∗*P < 0.05 versus control group; #P < 0.05 versus LPS + vehicle group; &P >0.05 versus LPS + vehicle group. TIM23, translocase of inner mitochondrial membrane 23 homolog; PGC-1*α*: peroxisome proliferator-activated receptor gamma, coactivator 1 alpha; mt-TFA, mitochondrial transcription factor A.

Measuring the mitochondrial mass is a quantitative method of monitoring the mitophagy. The level of the mitochondrial markers, TOM20 and TIM23, was used as indicators of mitophagy activity. The immunoblot analysis revealed that the level of TOM20 and TIM23 expression decreased following LPS exposure (P < 0.05 for Control versus LPS + vehicle). In contrast, Mdivi-1 treatment inhibited the downregulation of TOM20 and TIM23 expression (P < 0.05 for LPS + vehicle versus Mdivi-1; Figures 2(b) and 2(c)). This loss of mitochondrial mass may be due to mitophagy, or alternatively, it could be caused by an overall increase in autophagy or decreased mitochondrial biogenesis. Thus, we analyzed the expression level of two general autophagy-related proteins, LC3 and p62, as well as PGC-1*α* and mt-TFA, two mitochondrial biogenesis-related proteins. We found that the expression of PGC-1*α* and mt-TFA was not significantly downregulated following Mdivi-1 treatment (P > 0.05 for LPS + vehicle versus Mdivi-1; Figures 2(d) and 2(e)). Moreover, Mdivi-1 treatment did not significantly upregulate the level of LC3-II and downregulate that of P62, suggesting that autophagic flux was not robustly reinforced (P > 0.05 for LPS + vehicle versus Mdivi-1; Figures 2(f) and 2(g)). These data suggest that the effect of Mdivi-1 on mitochondrial mass is produced by inhibiting mitophagy. Therefore, it was confirmed that Mdivi-1 inhibited mitophagy activation in ALI.

### 3.3. Mdivi-1 Treatment Inhibits LPS-Induced Mitochondrial Dysfunction In Vitro

**Figure 3:**
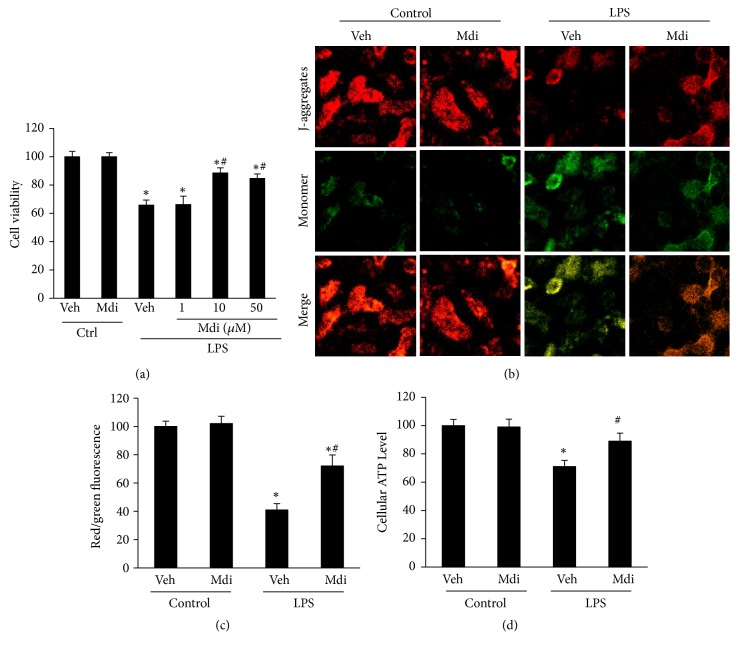
*Mdivi-1 treatment inhibits LPS-induced mitochondrial dysfunction in vitro*. (a) Quantification of the cells viability: the LPS + vehicle group showed remarkable decreased cell viability. Moderate (10*μ*M) and high (50 *μ*M) doses of Mdivi-1 treatment markedly reversed the cell viability induced by LPS exposure. (b) The cells were stained with JC-1 and the mitochondrial membrane potential (ΔΨm) was observed using laser confocal-scanning microscopy. Scale bar: 20 *μ*M. (c) Quantification of the intracellular red and green fluorescence of JC-1. (d) Quantification of the cellular ATP levels. Data are presented as the mean ± SD (n = 6 in each group). *∗*P < 0.05 versus the control group; #P < 0.05 versus the LPS + vehicle group.

To evaluate whether Mdivi-1 may act as a survival factor for lung alveolar epithelial cells, we examined the effect of Mdivi-1 on cell death induced by LPS insult. Cultured A549 cells with above-mentioned treatments, A549 cells viability significantly decreased in comparison with the control group after LPS stimulation (P < 0.05). Pretreated with moderate (10 *μ*M) and high (50 *μ*M ) doses of Mdivi-1 for 24h, the survival rates of A549 cells were markedly reversed, whereas a small (1 *μ*M) dose could not achieve such effects (P < 0.05 for LPS + vehicle versus LPS + 10 *μ*M and LPS + 50 *μ*M; P > 0.05 for LPS + vehicle versus LPS + 1 *μ*M; Figure 3(a)). So, we selected the concentration of 10 *μ*M of Mdivi-1 as the treatment in the following experiment* in vivo*.

To characterize the role of Mdivi-1 on LPS-induced mitochondrial dysfunction* in vitro*, mitochondrial membrane potential (ΔΨm) and cellular ATP levels were measured using commercial kits. As shown in Figure 3(b), it was found that ΔΨm was significantly decreased as indicated by the marked increases in green fluorescence when cells were exposed to LPS insult. However, when cells were pretreated with Mdivi-1, LPS-induced mitochondrial depolarization was significantly attenuated (P < 0.05 for LPS + vehicle versus Mdivi-1; Figure 3(c)). Furthermore, Mdivi-1 substantially restored mitochondrial ATP levels after LPS insult (P < 0.05 for LPS + vehicle versus Mdivi-1; Figure 3(d)).

### 3.4. Mdivi-1 Treatment Alleviates Lung Cell Apoptosis following LPS Administration in Rats

**Figure 4:**
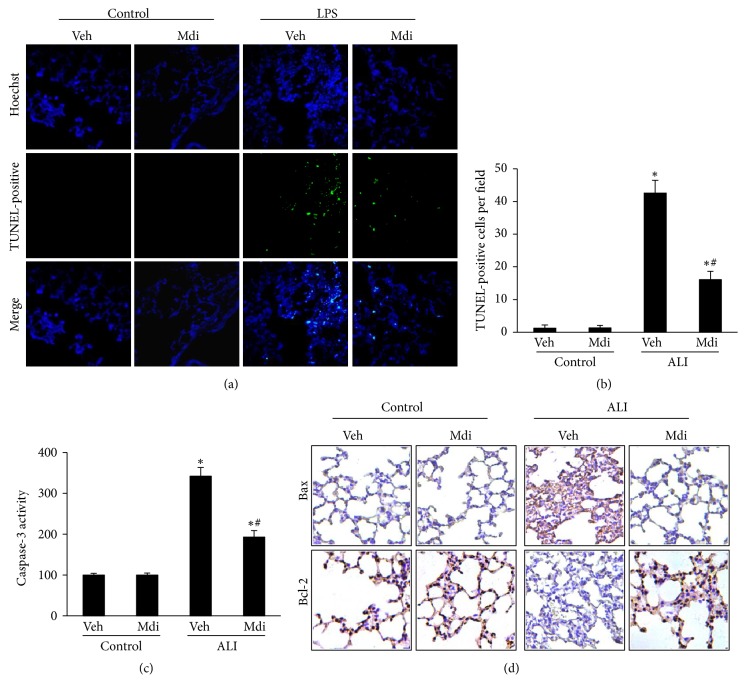
*Mdivi-1 prevents lung cell apoptosis in vivo*. (a) The level of pulmonary cell apoptosis increased following LPS administration, as determined by TUNEL staining (200× magnification). (b) TUNEL-positive cells were averaged over 10 microscopic fields per animal. LPS-challenged animals exhibited a significant increase in TUNEL-positive cells, which was reduced by treatment with Mdivi-1 treatment. (c) Caspase-3 activity in the lung tissues was elevated in the LPS group but suppressed in the group treated with Mdivi-1. (d) The level of Bax and Bcl-2 protein expression was determined by immunohistochemistry. The increased Bax expression and decreased Bcl-2 expression in the LPS group was reversed following Mdivi-1. Data are presented as the mean ± SD (n = 6 in each group). *∗*P < 0.05 versus control group; #P < 0.05 versus LPS + vehicle group.

Lung cell apoptosis was increased upon LPS administration, as determined by TUNEL staining. Treatment with Mdivi-1 was able to attenuate such LPS-induced cell death (P < 0.05 for LPS + vehicle versus Mdivi-1; Figures 4(a) and 4(b)). Similarly, the level of caspase 3 activity was also found to be elevated following LPS administration but was suppressed by Mdivi-1 treatment (P < 0.05 for LPS + vehicle versus Mdivi-1; Figure 4(c)). Moreover, we examined the effect of Mdivi-1 treatment on the regulation of Bcl-2 family protein expression. As shown in Figure 4(d), the immunohistochemical staining revealed that Mdivi-1 treatment significantly prevented the LPS-induced downregulation of Bcl-2 and upregulation of Bax protein expression (P < 0.05 for LPS + vehicle versus Mdivi-1; Figure 4(d)). Taken together, these findings indicate that Mdivi-1 protects the lung against LPS-induced apoptosis.

### 3.5. Mdivi-1 Treatment Protects Oxidative Stress in the Lung

**Figure 5:**
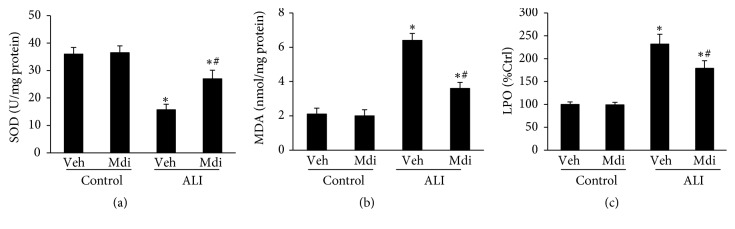
*Mdivi-1 ameliorates oxidative stress in pulmonary tissues*. (a) LPS administration induced significant SOD consumption, which was attenuated by Mdivi-1 treatment. (b) LPS administration induced significant MDA accumulation, which could be attenuated by Mdivi-1 treatment. (c) LPO levels in the lung increased in the LPS group but decreased in the group that received Mdivi-1. Data are presented as the mean ± SD (n = 6 in each group). *∗*P < 0.05 versus control group; #P < 0.05 versus LPS + vehicle. SOD, superoxide dismutase; MDA, malondialdehyde; LPO, Lipid peroxides.

Since oxidative stress is a leading cause of ALI pathogenesis, we investigated the level of SOD, MDA, and LPO in the lung tissues of LPS-exposed rats. As shown in Figure 5, the activity of SOD was highly reduced, whereas the level of MDA and LPO was increased following LPS administration (P < 0.05 for Control versus LPS + vehicle). However, treatment with Mdivi-1 markedly attenuated the consumption of SOD, as well as MDA and LPO accumulation (P < 0.05 for LPS + vehicle versus Mdivi-1; Figure 5), suggesting that Mdivi-1 provides protection against LPS-induced oxidative stress.

### 3.6. Mdivi-1 Treatment Prevents Inflammation Response in the Lung

**Figure 6:**
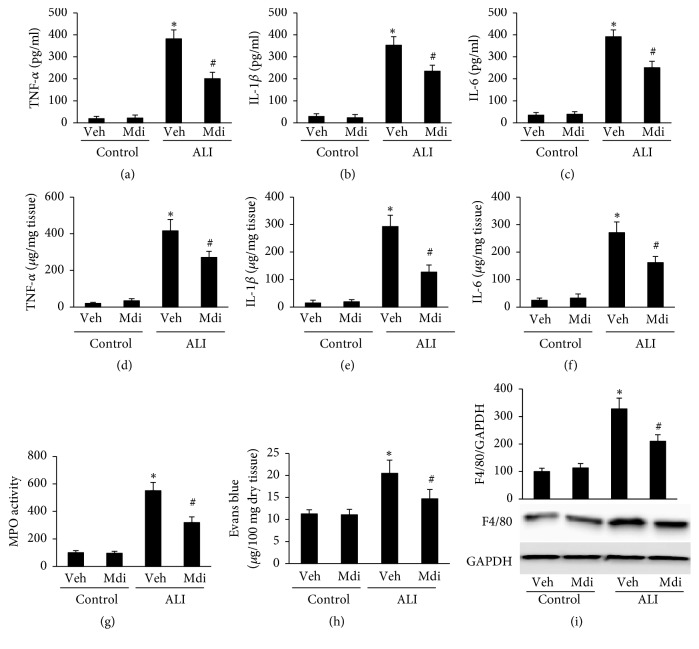
*Mdivi-1 downregulated the levels of proinflammatory cytokines as well as the lung inflammatory cells recruitment.* The levels of inflammatory mediators were elevated in the LPS + vehicle group, but decreased with Mdivi-1 treatment group. (a to c) TNF-*α*, IL-1*β* and IL-6 level in BALF. (d to f) TNF-*α*, IL-1*β* and IL-6 level in lung. (g) Lung MPO activity. (h) Evans Blue (EB) content in lungs. (i) Images of western blot result and semiquantitative analysis of F4/80 level. Data are presented as the mean ± SD (n = 6 in each group). *∗*P < 0.05 versus control group; #P < 0.05 versus LPS + vehicle.

Considering the key role of inflammatory mediators in ALI[4], we next measured the level of inflammatory mediators in the lung and BALF. As shown in Figure 6, increased levels of TNF-*α*, IL-1*β*, and IL-6 in the lungs and BALF of LPS-challenged rats were detected compared to those in the control group (P < 0.05 for Control versus LPS). However, Mdivi-1 treatment significantly suppressed the levels of above cytokines in the lung and BALF (P < 0.05 for LPS + vehicle versus LPS + Mdivi-1; Figures 6(a)–6(f)). Neutrophilic inflammation is associated with ALI[16]. Therefore, as an indicator of neutrophil infiltration, the lung MPO activity was detected. As shown in Figure 6(g), the lung MPO activity in LPS-challenged rats dramatically increased (P < 0.05 for Control versus LPS), and it was inhibited by Mdivi-1 treatment (P < 0.05 for LPS + vehicle versus LPS + Mdivi-1). In addition, the level of F4/80 (indicating macrophages) expression increased following LPS administration (P < 0.05 for Control versus LPS + vehicle), and Mdivi-1 treatment inhibited the upregulation of F4/80 expression (Figure 6(i)). These results suggest that Mdivi-1 treatment downregulates LPS-induced inflammation and resists inflammatory cells (neutrophils and macrophages) recruitment in lung.

## 4. Discussion

In the present study, we show that Mdivi-1 attenuated mitophagy and ALI induced by LPS using a rat model. Moreover, we demonstrated this protective role of Mdivi-1 to be mediated by ameliorating mitochondrial dysfunction, oxidative stress, apoptosis, and inflammation. These findings suggest that mitophagy may be involved in the pathogenesis of ALI, and inhibition of mitophagy may be a potential target for ALI therapy.

As previously described, a rat model of ALI was generated by delivering an intratracheal injection of LPS (10 mg/kg)[17–19]. As expected, remarkable lung injury and dysfunction was observed following LPS administration, as evidenced by the level of histopathologic deterioration, elevated W/D weight ratio, and decreased oxygenation index, which is similar to that reported in previous studies [2, 20]. Then, we investigated the protective effects of different doses of Mdivi-1 (low: 1 mg/kg; moderate: 3 mg/kg; high: 5 mg/kg) against LPS-induced ALI. Treatment with the moderate and high dose of Mdivi-1 was associated with beneficial effects in LPS-induced ALI, as reflected by an improvement in pathological changes, water content in the lungs, and oxygenation index.

A growing number of reports suggest that mitophagy is relevant to several human diseases. The study by Mizumura et al. found that excessive mitophagy promotes mitochondrial damage in chronic obstructive pulmonary disease [9]. In addition, Wu et al. showed excessive autophagy and mitophagy activation in blood-brain barrier disruption, cell death, and oxidant stress induced by traumatic brain injury [21]. While growing evidence indicates that mitophagy may be enhanced in sepsis [9, 22, 23], the role of mitophagy in ALI remains unknown. Mitophagy leads to the phagocytosis of mitochondria, thus decreasing the intracellular mitochondrial mass when it is enhanced [24]. In the present study, we investigated changes in the mitochondrial mass to detect mitophagy. We found that TOM20 and TIM23 levels were decreased in rats with ALI, indicating that mitophagy was upregulated. Previous studies use Mdivi-1, a pharmacological Drp1 inhibitor, to investigate the effects of inhibiting mitophagy [25]. Consistent with such studies, we found that Mdivi-1 treatment inhibited the downregulation of TOM20 and TIM23 expression. In addition, the decrease in mitochondrial mass may result from the reduction of mitochondrial production or an increase in autophagy. We found that the increased LC3 II/LC3 I ratio and decreased p62 expression induced by LPS were not reversed following Mdivi-1 treatment. Moreover, expression of the mitochondrial biogenesis markers, PGC-1*α* and mt-TFA, was not significantly downregulated following LPS or Mdivi-1 treatment. These findings indicted that Mdivi-1 had no significant effect on mitochondrial production or general autophagy, suggesting that Mdivi-1 affects mitochondrial mass by modulating mitophagy.

Mitochondrial dysfunction is correlated with sepsis, and normal depolarization of the mitochondrial membrane is vital to cell survival [26]. Opening of the mitochondrial permeability transition pore (mPTP) causes dissipation of mitochondrial membrane potential (ΔΨm) and inhibition of ATP production and results in mitochondria dysfunction and even cell death. In the current study, decreased cell viability, collapsed ΔΨm, and low intracellular ATP developed in A549 cells with LPS insult, however, Mdivi-1 prevented this mitochondrial injury and cell death* in vitro*. Several studies have shown that mitochondrial dynamics are linked to mitochondrial quality control by mitophagy, and Mdivi-1 alleviates the mitochondrial damage in traumatic brain injury[21], doxorubicin-induced cardiotoxicity [27], and triptolide-induced hepatotoxicity.[28] Similarly, our results demonstrate that Mdivi-1 treatment attenuated lung injury, indicating that the inhibition of mitophagy may play a protective role in ALI.

Apoptosis is a widely accepted critical pathophysiological process in ALI [29, 30]. Thus, the results of the present study further demonstrate that Mdivi-1 treatment provided protection in ALI by functioning as an inhibitor of apoptosis. In this study, we observed a significant reduction in the density of apoptotic cells after delivering Mdivi-1 treatment. In addition, the change in the expression of several apoptosis-related proteins (i.e., Bcl-2, Bax, and caspase 3) was assessed. It has been well-established that Bcl-2 is an antiapoptotic protein, whereas Bax is considered to be a proapoptotic member of the Bcl-2 family proteins [31–33]. In our study, Mdivi-1 inhibited the LPS-induced upregulation of Bax downregulation of Bcl-2 and activation of caspase 3. Collectively, these findings suggest that Mdivi-1 treatment plays an antiapoptosis role in LPS-induced ALI.

Oxidative stress also has a critical role in the pathophysiology associated with sepsis. [8, 29] In the present study, superoxide dismutase (SOD) consumption, lipid peroxides (LPO) levels, and malondialdehyde (MDA) accumulation were used as a measure of oxidative stress. Increased LPO levels, MDA accumulation, and SOD consumption in the lung tissues of LPS-challenged rats were all reversed by Mdivi-1 treatment. These results suggest that Mdivi-1 treatment can significantly reduce LPS-induced oxidative stress in ALI.

Proinflammatory cytokines play an essential role in the initiation and amplification of inflammatory responses [34, 35], and recruitment of neutrophils is a key event in development of ALI. The effect of neutrophils is supported by studies where lung injury is reversed by neutrophils consumption [36, 37]. In the present study, rats exposed to LPS exhibited a significant upregulation of inflammatory mediators and recruitment of inflammatory cells including neutrophils and macrophages; we found that Mdivi-1 treatment dramatically prevented the increase of proinflammatory cytokines (TNF-*α*, IL-1*β*, and IL-6) in lung and BALF, MPO activity, and level of F4/80. These results demonstrate that Mdivi-1 treatment ameliorates LPS-induced lung inflammation response.

## 5. Conclusion

The present study demonstrates that the mitophagy inhibitor, Mdivi-1, inhibits mitophagy and protects ALI-challenged rats from LPS-induced apoptosis, oxidative stress, and inflammation. Furthermore, in this study, the wider protection and inhibition of mitophagy provided by Mdivi-1 (3 or 5 mg/kg) may represent a promising target for the treatment of ALI in the future.

## Data Availability

All data supporting for this study is found in the manuscript or is available upon request from the correspinding author, Youtan Liu.
